# Plasma Uric Acid Levels Correlate with Inflammation and Disease Severity in Malian Children with *Plasmodium falciparum* Malaria

**DOI:** 10.1371/journal.pone.0046424

**Published:** 2012-10-05

**Authors:** Tatiana M. Lopera-Mesa, Neida K. Mita-Mendoza, Diana L. van de Hoef, Saibou Doumbia, Drissa Konaté, Mory Doumbouya, Wenjuan Gu, Karim Traoré, Seidina A. S. Diakité, Alan T. Remaley, Jennifer M. Anderson, Ana Rodriguez, Michael P. Fay, Carole A. Long, Mahamadou Diakité, Rick M. Fairhurst

**Affiliations:** 1 Laboratory of Malaria and Vector Research, National Institute of Allergy and Infectious Diseases, National Institutes of Health, Bethesda, Maryland, United States of America; 2 Departamento de Biomedicina Molecular, Centro de Investigación y Estudios Avanzados, México City, México; 3 Department of Microbiology, New York University School of Medicine, New York, New York, United States of America; 4 Malaria Research and Training Center, Faculty of Medicine, Pharmacy and Odontostomatology, University of Bamako, Bamako, Mali; 5 SAIC-Frederick, Inc., NCI-Frederick, Frederick, Maryland, United States of America; 6 Biostatistics Research Branch, National Institute of Allergy and Infectious Diseases, National Institutes of Health, Rockville, Maryland, United States of America; 7 Department of Laboratory Medicine, NIH Clinical Center, National Institutes of Health, Bethesda, Maryland, United States of America; The George Washington University Medical Center, United States of America

## Abstract

**Background:**

*Plasmodium falciparum* elicits host inflammatory responses that cause the symptoms and severe manifestations of malaria. One proposed mechanism involves formation of immunostimulatory uric acid (UA) precipitates, which are released from sequestered schizonts into microvessels. Another involves hypoxanthine and xanthine, which accumulate in parasitized red blood cells (RBCs) and may be converted by plasma xanthine oxidase to UA at schizont rupture. These two forms of ‘parasite-derived’ UA stimulate immune cells to produce inflammatory cytokines *in vitro*.

**Methods and Findings:**

We measured plasma levels of soluble UA and inflammatory cytokines and chemokines (IL-6, IL-10, sTNFRII, MCP-1, IL-8, TNFα, IP-10, IFNγ, GM-CSF, IL-1β) in 470 Malian children presenting with uncomplicated malaria (UM), non-cerebral severe malaria (NCSM) or cerebral malaria (CM). UA levels were elevated in children with NCSM (median 5.74 mg/dl, 1.21-fold increase, 95% CI 1.09–1.35, n = 23, p = 0.0007) and CM (median 5.69 mg/dl, 1.19-fold increase, 95% CI 0.97–1.41, n = 9, p = 0.0890) compared to those with UM (median 4.60 mg/dl, n = 438). In children with UM, parasite density and plasma creatinine levels correlated with UA levels. These UA levels correlated with the levels of seven cytokines [IL-6 (r = 0.259, p<0.00001), IL-10 (r = 0.242, p<0.00001), sTNFRII (r = 0.221, p<0.00001), MCP-1 (r = 0.220, p<0.00001), IL-8 (r = 0.147, p = 0.002), TNFα (r = 0.132, p = 0.006) and IP-10 (r = 0.120, p = 0.012)]. In 39 children, UA levels were 1.49-fold (95% CI 1.34–1.65; p<0.0001) higher during their malaria episode [geometric mean titer (GMT) 4.67 mg/dl] than when they were previously healthy and aparasitemic (GMT 3.14 mg/dl).

**Conclusions:**

Elevated UA levels may contribute to the pathogenesis of *P. falciparum* malaria by activating immune cells to produce inflammatory cytokines. While this study cannot identify the cause of elevated UA levels, their association with parasite density and creatinine levels suggest that parasite-derived UA and renal function may be involved. Defining pathogenic roles for parasite-derived UA precipitates, which we have not directly studied here, requires further investigation.

**Trial Registration:**

ClinicalTrials.gov NCT00669084

## Introduction

Each year, *Plasmodium falciparum* causes an estimated 655 million episodes of malaria worldwide and 1 million deaths, mostly in young children living in sub-Saharan Africa [1] [2]. A better understanding of malaria pathogenesis is essential to improve the survival of children with severe malaria who often die despite the prompt administration of supportive measures and effective antimalarial drugs. The pathogenesis of *P. falciparum* malaria is complex, involving multiple parasite and human factors that, in combination, produce varying levels of immune stimulation and microvascular inflammation [3]–[5]. While the degree of inflammation generally correlates with the severity of a malaria episode, the parasite factors that elevate host inflammatory responses from beneficial to pathological levels are not well characterized. Only a few *P. falciparum*-derived factors have been shown to activate immune cells to produce the inflammatory responses associated with malaria. These include glycosylphosphatidylinositol (GPI) anchors and DNA-laden hemozoin (a polymer of heme moieties derived from digested hemoglobin), which are released into circulation when sequestered *P. falciparum*-infected red blood cells (RBCs) rupture in microvessels [5]–[8]. These two parasite factors interact with Toll-like receptors (TLRs) on immune cells *in vitro* to elicit some of the same cytokine responses associated with human malaria syndromes.

Uric acid (UA) is produced in humans and higher primates as the final product of purine metabolism [9]. Its biosynthesis is catalyzed by xanthine oxidase, which produces reactive oxygen species (ROS) as by-products. Three recent studies have implicated UA as an additional parasite-derived factor that may contribute to malaria pathogenesis. In the first study, Orengo *et al.* showed that soluble UA and ROS, derived from the degradation of hypoxanthine and xanthine accumulated in *P. yoelii*–infected RBCs, activate murine dendritic cells *in vitro* to produce TNFα [10]. In the second study, the production of cytokines (IL-1β, IL-6, TNFα and IL-10) by human peripheral blood mononuclear cells (PBMCs) stimulated with *P. falciparum*-infected RBCs was significantly reduced in the presence of allopurinol (an inhibitor of xanthine oxidase) or uricase (an enzyme which degrades UA) [11]. Since *P. falciparum* requires hypoxanthine and xanthine for *de novo* synthesis of purines [12], [13], it was proposed that these accumulated precursors are released at schizont rupture and converted to UA by plasma xanthine oxidase. In microvessels where parasitized RBCs sequester *en masse* and may rupture synchronously, transient high levels of soluble UA may directly stimulate PBMCs. In the third study, van de Hoef *et al.* found that UA precipitates accumulate in the cytosol and parasitophorous vacuole of intraerythrocytic parasites as they mature [14]. These UA precipitates are released from the parasite at schizont rupture and activate human dendritic cells *in vitro*. Whether the inflammatory potential of these parasite-derived UA precipitates *in vivo* is similar to that of UA crystals, which cause gout [15], has not been investigated.

We hypothesized that UA contributes to the pathology of human malaria by stimulating the production of cytokines from immune cells. To explore this hypothesis, we measured plasma UA levels in Malian children with *P. falciparum* malaria and correlated them with parasite densities, plasma creatinine levels (as a measure of renal function), disease severity and plasma cytokine levels. We found that UA levels (i) increase during episodes of uncomplicated malaria; (ii) increase further during episodes of severe malaria; (iii) correlate with parasite densities and creatinine levels; and (iv) correlate with levels of seven cytokines associated with disease severity in our patient population. These data support a model of malaria pathogenesis in which elevated levels of UA, resulting in part from the combined effects of rupturing *P. falciparum*-infected RBCs and subclinical renal insufficiency, stimulate the production of inflammatory cytokines.

## Methods

### Ethics statement

All protocol activities were approved by the Ethics Committee of the Faculty of Medicine, Pharmacy and Odontostomatology at the University of Bamako, Mali, and the Institutional Review Board of the National Institute of Allergy and Infectious Diseases at the National Institutes of Health, United States. The parent or guardian of each child gave written informed consent. The protocol is registered at clinicaltrials.gov (NCT00669084).

### Study site and participants

To evaluate the effects of human genetic polymorphisms on the incidence of falciparum malaria, we enrolled 1257 children into a prospective longitudinal cohort study in May 2008. Nearly all children aged 6 months–17 years from three neighboring villages (Kenieroba, Fourda and Bozokin) participated. In these villages, located approximately 75 km southwest of Bamako, *P. falciparum* transmission is seasonal (June–December) and intense. Only children with treatment-seeking behavior for symptoms of malaria were evaluated for the disease; that is, no active case detection was conducted. We used the findings from history taking and physical examination, along with measurements of hemoglobin and glucose (Hemocue®, Hemocue AB, Angelholm, Sweden), to diagnose each child with uncomplicated or severe malaria. Parasite densities were quantified from thick blood films by counting the number of ring-stage parasites until 300 leukocytes were also counted, then multiplying this number by 25 (which assumes an average number of 7500 leukocytes per µl whole blood). Hemoglobin levels were measured again 72 hours later.

Uncomplicated falciparum malaria was defined by fever (axillary temperature ≥37.5°C, or history of fever in the previous 24 hours) in the presence of any asexual *P. falciparum* density with no symptoms or signs of severe malaria or other etiologies of febrile illness discernible by history taking and physical examination. We treated these children with artesunate 4 mg/kg plus amodiaquine 10 mg/kg, given orally, for 3 consecutive days (days 0, 1, and 2) and confirmed they were aparasitemic on day 3. Children with uncomplicated malaria and *P. falciparum* density ≥100,000/µl whole blood were treated as for severe malaria.

Severe *P. falciparum* malaria was defined by the presence of any parasite density plus any one of the following: coma (defined as Blantyre coma score ≤2), convulsions (witnessed by the study investigator), severe prostration, severe anemia (hemoglobin ≤5 g/dl), respiratory distress (deep acidotic breathing), hypoglycemia (serum glucose ≤40 mg/dl), jaundice/icterus, shock (systolic blood pressure ≤50 mmHg, rapid pulse, cool extremities), cessation of eating and drinking, and repetitive vomiting. All children diagnosed with cerebral malaria met the criterion of coma, had neither hypoglycemia nor convulsions, and responded clinically to antimalarial therapy alone; ocular fundoscopy was not performed. Children with severe malaria were treated with artesunate, given intravenously, followed by the drug regimen for uncomplicated malaria when the child was able to take oral medication.

### Plasma collection

In May 2008, before the malaria season, we obtained 2–10 ml venous blood from a sub-cohort of 162 children aged 2–14 years who were healthy and aparasitemic (thick blood film negative for *P. falciparum*). During the 2008 malaria season, we obtained 2–10 ml venous blood from all children in the cohort who developed malaria, at each episode and prior to antimalarial drug administration. We collected all blood samples into sodium heparin Vacutainers® (Becton-Dickinson, Franklin Lakes, NJ), separated plasma by centrifugation (2500 rpm×10 min) within 4–6 hours of the blood draw, and immediately stored aliquots at −80°C until use.

### Measurement of cytokine levels

Plasma samples were thawed at ambient temperature and centrifuged (14,000 rpm×10 min) at 4°C. Using polystyrene bead-based multiplex assay kits (Invitrogen Corp., Carlsbad, CA) according to the manufacturer's instructions, we quantified the levels of IL-6, IL-10, sTNFRII, MCP-1, IL-8, TNFα, IP-10, IFNγ, GM-CSF and IL-1β. We tested samples at a 1∶3 dilution. We read the plates using a Luminex LX100 instrument (Luminex Corp., Austin, TX) and interpolated the results from five-parameter-fit standard curves generated by Xponent 3.1 software (Luminex Corp.). We changed all cytokine or chemokine values = “0.0” to half the next largest value. We replaced IP-10 values = “>380” with the median of all the rest greater than 380 (median = 560), and changed all cytokine values “<X” to “X/2”.

### Measurement of uric acid levels

We tested plasma samples in triplicate using QuantiChrom™ Uric Acid Assay Kit (Bioassay Systems, Hayward, CA). This method utilizes 2,4,6-tripyridyl-s-triazine which forms a blue-colored complex specifically with iron in the presence of UA. The colorimetric reaction was read at 590 nm using a plate reader (Perkin Elmer, Waltham, MA). The method has a linear detection range between 0.22–30 mg/dl (13–80 µM) UA. We used the mean of triplicate values for UA levels in subsequent analyses.

### Measurement of creatinine levels

Plasma creatinine levels were determined using the Enzymatic Creatinine Flex® Reagent cartridge (Siemens Healthcare Diagnostics, Inc., Newark, DE). In this method, hydrogen peroxide and chromogens (4-aminophenazone and 2,4,6-triiodo-3-hydroxybenzoic acid) form an amount of colored end product that is proportional to the amount of creatinine in the sample. The colored end product was measured at 540 and 700 nm using the Dimension Vista® System (Siemens Healthcare Diagnostics, Inc.). The analytical measurement range of this method in plasma is 0.14–20 mg/dl.

### Statistical analysis

We compared the values for continuous variables (age, parasite density, hemoglobin level, creatinine level, UA level and cytokine level) between groups of children with uncomplicated and cerebral or non-cerebral severe malaria using the Mann-Whitney test with the associated fold-change confidence intervals calculated using the Hodges-Lehmann method on the log-transformed values. The geometric mean titers of UA and creatinine in paired plasma samples were compared using the Paired t test on log-transformed values. We evaluated the relationship between log_10_ UA levels, log_10_ parasite densities, log_10_ cytokine levels and log_10_ creatinine levels by Pearson's correlation coefficient, and tested whether they were different from 0 using the t-distribution. To determine how parasite density and creatinine level predict UA level, we used a linear model with log_10_ UA level as a response. Analyses were done in either GraphPad version 5.01 (GraphPad Software, La Jolla, CA) or R version 2.13.0 (R Core Development Team, http:/www.r-project.org).

## Results

Parasite-derived UA stimulates the production of inflammatory cytokines from human PBMCs *in vitro* [11]. To explore whether this stimulation may occur *in vivo*, we measured plasma UA levels, parasite densities and plasma cytokine levels in a cohort of Malian children who developed *P. falciparum* malaria. Plasma samples were available for 90% (501/557) of children who developed a first episode of malaria in 2008. Of these samples, 473, 24 and 4 were from children with uncomplicated malaria (UM), non-cerebral severe malaria (NCSM) and cerebral malaria (CM), respectively. We included in this study an additional 5 plasma samples from children who developed CM in the 2009 malaria season, giving a total of 9 CM samples.

**Table 1** shows the characteristics of these children, stratified by clinical presentation. As expected, children with CM were younger (all were ≤3 years) than those with UM (p<0.0001). Compared to children with UM, median parasite densities were higher in children with NCSM (p = 0.0048) and CM (p = 0.0060). Median hemoglobin levels at presentation did not differ between children with UM, NCSM and CM. The degree of anemia, however, worsened in all three groups of children after 72 hours of antimalarial therapy, and was significantly more severe in those children with CM (p = 0.0001) (**Table 1**). Compared to children with UM, those with NCSM and CM had higher and lower levels of plasma creatinine, respectively, but only the latter difference was statistically significant (**Table 1**).

**Table 1:** Demographic, parasitological and biochemical parameters, stratified by clinical presentation.^a^

Parameter^b^	UM	NCSM^c^	Fold-change	p-value^d^	CM	Fold-change	p-value^d^
	n = 473	n = 24	(95% CI)	NCSM *vs.* UM	n = 9	(95% CI)	CM *vs.* UM
			NCSM/UM			CM/UM	
**% males**	49.7	54.2	1.20^e^	0.6823^f^	33.3	0.51^e^	0.5040^f^
			(0.52–2.89)			(0.11–2.17)	
**Age (years)**	5	6	1.00	0.6672	1	0.25	<0.0001
	(3–9)	(3–8)	(0.80–1.50)		(0.875–2)	(0.17–0.40)	
**Parasite density (/µl)**	8600	22888	3.00	0.0048	34350	5.77	0.0060
	(787.5–26150)	(6975–46012)	(1.38–6.92)		(10300–138150)	(1.70–22.7)	
**Hb level at 0 hours (g/dl)**	10.5	11.0	1.03	0.4976	9.0	0.91	0.2270
	(9.3–11.6)	(8.9–12.2)	(0.95–1.10)		(7.6–12.0)	(0.77–1.06)	
**Hb level at 72 hours (g/dl)**	10.2	9.9	0.94	0.1019	7.5	0.76	0.0001
	(9.1–11.4)	(8.4–10.8)	(0.87–1.01)		(6.7–9.1)	(0.67–0.86)	
	n = 451						
**Creatinine (mg/dl)**	0.344	0.412	1.15	0.0702	0.250	0.78	0.0302
	(0.281–0.423)	(0.289–0.484)	(0.99–1.34)		(0.222–0.351)	(0.63–0.98)	
	n = 466	n = 23			n = 8		
**Uric acid (mg/dl)**	4.60	5.74	1.21	0.0007	5.69	1.19	0.0890
	(3.84–5.39)	(4.67–6.86)	(1.09–1.35)		(4.30–6.70)	(0.97–1.41)	
	n = 438	n = 23					

a UM = uncomplicated malaria; NCSM = non-cerebral severe malaria; CM = cerebral malaria.

b The median (IQR) of each parameter is shown, unless otherwise indicated.

c The 24 children met the following criteria for NCSM: 1 had severe malarial anemia, 9 had prostration, and 14 had repetitive vomiting.

d p-values were calculated using the Mann-Whitney test, unless otherwise indicated.

e For sex comparisons, the associated statistic is the odds ratio (instead of fold-change). Matching 95% CIs were calculated as described [38].

f p-values were calculated using the Fisher's exact test.

**Figure 1:**
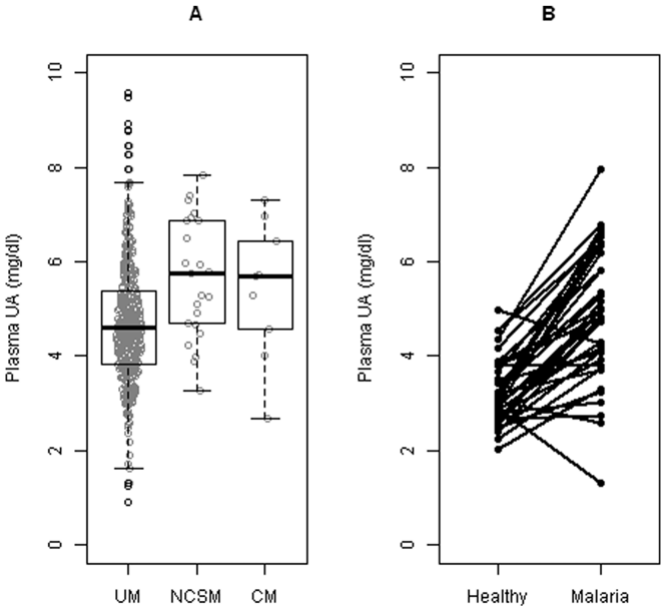
Uric acid (UA) elevations in Malian children with uncomplicated and severe falciparum malaria. **a,** Plasma UA levels were measured in Malian children who presented with uncomplicated (UM), non-cerebral severe (NCSM) and cerebral malaria (CM). We show traditional boxplots (i.e., middle line is median, box is interquartile range) with points randomly jittered according to their density similar to violin plots [36] [37]. **b,** Plasma UA levels were measured in a cohort of 39 healthy aparasitemic Malian children in May 2008 (prior to the malaria season) and again at their first episode of UM during the 2008 malaria season.

To determine whether plasma UA levels were associated with malaria severity, we compared them in groups of children that differed in clinical presentation. We found that UA levels were increased in children with NCSM (median 5.74 mg/dl, 1.21-fold increase, 95% CI 1.09–1.35, p = 0.0007) and CM (median 5.69 mg/dl, 1.19-fold increase, 95% CI 0.97–1.41, p = 0.0890) compared to those in children with UM (median 4.60 mg/dl) (**Table 1** **and** **Fig. 1a**). Among these 501 children, we identified 39 children who provided a ‘baseline’ plasma sample in May 2008 when they were healthy and aparasitemic. In these paired plasma samples, we found that baseline UA levels increased 1.49-fold (95% CI 1.34, 1.65; p<0.0001) during a child's malaria episode (GMT 3.14 mg/dl *vs.* 4.67 mg/dl) (**Fig. 1b**).

To explore the cause of elevated UA levels during a malaria episode, we performed a series of univariate analyses on data obtained from children with UM. We found that log_10_ UA levels correlated significantly with log_10_ parasite densities (Pearson r = 0.1641, n = 438, p = 0.0006) but not age (r = −0.0144, n = 438, p = 0.7631). Since UA levels are known to be affected by renal function (which may be compromised during a malaria episode) [9], [16], [17], we also measured plasma creatinine levels. We found that log_10_ UA levels correlated significantly with log_10_ creatinine levels (r = 0.1634, n = 433, p = 0.0006). In the 39 baseline plasma samples, we found that baseline creatinine levels increased 1.13-fold (95% CI 1.05, 1.22; p = 0.003) during a malaria episode (GMT 0.352 mg/dl *vs.* 0.398 mg/dl). While these data support the possibility that parasite-derived UA contributes to the elevated UA levels in children with UM, they suggest that subclinical renal insufficiency may contribute as well.

To investigate a potential role for UA in malaria pathogenesis, we first measured the levels of ten cytokines (IL-6, IL-10, MCP-1, sTNFRII, IL-8, IP-10, TNFα, IFNγ, GM-CSF and IL-1β) that other studies [18]–[23] have implicated in the development of severe malaria in humans. With the exception of IL-1β, the levels of all cytokines increased significantly with disease severity in our patient population (**Table 2**). We then explored whether UA may stimulate the production of these cytokines by performing a series of correlation analyses on the levels of UA and cytokines in children with UM – a clinically homogeneous group. We found positive correlations between log_10_ UA levels and the log_10_ levels of seven cytokines, listed here from strongest to weakest: IL-6 (Pearson r = 0.259, p<0.00001), IL-10 (r = 0.242, p<0.00001), sTNFRII (r = 0.221, p<0.00001), MCP-1 (r = 0.220, p<0.00001), IL-8 (r = 0.147, p = 0.002), TNFα (r = 0.132, p = 0.006) and IP-10 (r = 0.120, p = 0.012). These seven correlations remained significant at the 0.05 level even after correcting for the fact that we tested ten cytokines using Holm's multiple comparison correction [24]. No correlations were identified between UA and IFN-γ (r = 0.073, p = 0.131), GM-CSF (r = 0.048, p = 0.326) and IL-1β (r = 0.025, p = 0.603).

**Table 2:** Cytokine levels, stratified by clinical presentation.^a^

Cytokine^b^	UM	NCSM^c^	Fold-change	p-value^d^	CM	Fold-change	p-value^d^
	n = 473	n = 24	(95% CI)	NCSM *vs.* UM	n = 9	(95% CI)	
			NCSM/UM			CM/UM	CM *vs.* UM
**IL-6** e	22.8	340	11.1	<0.0001	312	14.1	<0.0001
	(7.08–96.4)	(120–616)	(5.62–21.7)		(130–968)	(4.49–41.1)	
	n = 471						
**IL-10**	134	1119	9.27	<0.0001	1726	14.9	<0.0001
	(20.6–605)	(498–1892)	(4.37–20.9)		(864–4304)	(4.53–54.3)	
	n = 471						
**MCP-1**	338	4784	10.9	<0.0001	1067	3.77	0.0067
	(139–969)	(1488–9854)	(6.11–19.3)		(497–5917)	(1.57–9.44)	
	n = 472						
**sTNFRII**	3.29	6.56	1.83	<0.0001	7.19	1.94	0.0003
	(1.93–5.07)	(5.36–7.59)	(1.47–2.34)		(4.74–8.32)	(1.40–2.91)	
	n = 467						
**IL-8**	66.0	268	3.41	<0.0001	91.8	2.05	0.1203
	(29.1–154)	(126–422)	(2.14–5.20)		(45–849)	(0.85–5.88)	
**TNFα**	1.3	4.3	3.56	<0.0001	3.6	3.25	0.0047
	(0.6–2.7)	(2.6–10.6)	(2.25–5.41)		(1.6–19.2)	(1.46–7.87)	
	n = 471						
**IP-10**	221	425	1.98	0.0008	815	3.76	0.0003
	(102–426)	(236–845)	(1.35–2.92)		(437–1609)	(2.01–7.04)	
	n = 470						
**IFNγ**	2.14	3.13	1.53	0.0504	5.23	2.92	0.0102
	(1.06–4.83)	(2.00–6.76)	(1.00–2.30)		(3.67–37.1)	(1.41–7.39)	
	n = 460						
**GM-CSF**	4.49	11.7	2.35	0.0006	11.3	1.60	0.2954
	(2.94–9.75)	(5.48–25.2)	(1.48–3.75)		(2.6–25.2)	(0.37–6.38)	
	n = 459				n = 4		
**IL-1β**	1.8	4.3	1.22	0.2668	4.3	2.06	0.1936
	(0.45–5.97)	(0.56–6.42)	(0.86–2.37)		(2.06–9.59)	(0.64–6.91)	
	n = 458						

a UM = uncomplicated malaria; NCSM = non-cerebral severe malaria; CM = cerebral malaria.

b The median (IQR) of each cytokine level is shown.

c The 24 children met the following criteria for NCSM: 1 had severe malarial anemia, 9 had prostration, and 14 had repetitive vomiting.

d p-values were calculated using the Mann-Whitney test.

e All cytokine concentrations are expressed as pg/ml, except for sTNFRII (ng/ml).

To explore how parasite density and creatinine level predict UA level, we used a linear model with log_10_ UA level as a response. The model predicts that for a fixed level of creatinine, there is a significant 1.06-fold (95% CI 1.03–1.10; p<0.0001) increase in UA for each log_10_ increase in parasite density. Further, for a fixed level of parasitemia, there is a 1.50-fold (95% CI 1.25–1.81, p<0.0001) increase in UA for each log_10_ increase in creatinine.

## Discussion

Here we report data from a large number of Malian children who developed uncomplicated or severe malaria in the 2008 malaria season. We found that baseline plasma UA levels increase in UM and further increase in NCSM and CM. In children with UM, UA levels correlated with parasite densities and creatinine levels, suggesting that parasite-derived UA and subclinical renal insufficiency contribute in part to elevating UA levels. UA levels also correlated with IL-6, IL-10, sTNFRII, MCP-1, IL-8, TNFα and IP-10 levels, all of which were elevated in children with severe malaria. These data suggest a model of malaria pathogenesis in which elevated levels of UA stimulate immune and possibly other host cells to produce excessive levels of inflammatory cytokines.

Studies of non-malaria diseases have shown that crystalline forms of UA are proinflammatory, and have begun to elucidate the mechanisms by which they induce inflammation. In gout, for example, UA crystals have been shown to induce IL-1β production through the NALP3 inflammasome pathway [15], [25]; whether *P. falciparum*-derived UA precipitates activate immune cells via this mechanism has not yet been investigated. Recent work suggests that UA functions as a danger signal to the immune system when it is released from necrotic cells [26], [27] and mediates the adjuvant effect of alum [28]. How UA activates immune (and non-immune) cells, however, is incompletely understood [29], [30]. Parasite-derived UA precipitates have also been observed in *P. falciparum* and *P. vivax*-infected RBCs obtained directly from Peruvian patients with *P. falciparum* and *P. vivax* malaria [14]. If elevated UA levels are achieved in *P. vivax* malaria, they may also contribute to the pathogenesis of this disease, for example, by stimulating the production of cytokines that induce fever and dyserythropoiesis. Elucidating the pathways by which *Plasmodium*-derived soluble and precipitated UA stimulate host cells may improve our understanding of the pathogenesis of other diseases in which elevated levels of soluble or insoluble UA are present [31].

Our findings not only support the hypothesis that UA contributes to the pathogenesis of *P. falciparum* malaria in African children, but also raises the possibility that the UA level may serve as a useful biomarker for severe disease. In addition, our findings may help to explain those of Sarma *et al.* [32], who showed that the co-administration of allopurinol and quinine more effectively reduced inflammation (as measured by fever clearance rate) than quinine alone in a study of Indian adults with severe *P. falciparum* malaria. Our data also provide some evidence to support the need for clinical trials to investigate whether allopurinol, which has been safely administered at UA-lowering doses to patients with severe *P. falciparum* malaria [32], might be useful as an adjunctive treatment for severe malaria syndromes that kill African children. Whether uricosuric drugs (e.g., probenecid, benzbromorone and sulfinpyrazone) might benefit such patients also merits investigation.

This study reports data from a relatively large number of Malian children of all ages who presented with malaria syndromes that were clinically well-defined. To our knowledge, this is the first study to specifically investigate associations between UA and inflammatory cytokines during episodes of human malaria. Before making these correlations, we confirmed that the cytokines we measured increase with disease severity, thus implicating them in the pathogenesis of severe malaria in our Malian study population. Previous studies that measured UA levels in patients with malaria tested the hypothesis that UA is an indirect marker of oxidative stress [33]–[35]. This is because the formation of UA from hypoxanthine and xanthine generates ROS. Only two previous studies examined the relationship between UA levels and *P. falciparum* densities in patients with malaria. Bertrand *et al*. [33] describe a weak correlation (r = 0.06, p>0.05) in a group of 60 Cameroonian adults with UM. In comparing groups of Nigerian children with asymptomatic parasitemia, UM and severe malaria, Iwalokun *et al.* [35] showed that the association between UA levels and parasite density gets stronger with disease severity; however, this correlation (r = 0.61, p<0.05) was significant only in the group of severe cases. Our analysis of 438 Malian children with UM shows a moderate, but highly significant, correlation (r = 0.1641, p = 0.0006) between UA levels and parasite densities.

Our study has several limitations. First, we are unable to identify the cause of elevated UA levels in our patients. During a malaria episode, excess soluble UA may be produced by a variety of processes, including the dissolution of parasite-derived UA precipitates, the conversion of parasite-accumulated hypoxanthine and xanthine to UA by plasma xanthine oxidase, and the hemolysis of both parasitized and non-parasitized RBCs. The levels of UA produced by any of these processes may correlate with parasite density. More detailed studies of renal function in children with malaria are needed to determine whether subclinical renal insufficiency also helps to increase the concentration of UA in plasma. Second, we are unable to quantify the ‘local’ levels of parasite-derived UA in microvessels. UA and cytokine levels may be considerably higher in the post-capillary venules where schizonts rupture than in the large veins from which we obtain plasma. We are also unable to quantify the amount of parasite-derived UA precipitates that may be present as un-dissolved, yet immunostimulatory, material in microvessels. Third, the correlations between UA and cytokine levels cannot definitively establish that UA is directly stimulating immune cells to produce the cytokines we measured. In support of this possibility, however, we found that UA levels correlate significantly with IL-6, TNFα and IL-10 levels. These findings are consistent with those of Orengo *et al.* [10], [11] who found that parasite-derived UA directly stimulates human PBMCs to produce IL-6, TNFα and IL-10 *in vitro*.

In summary, the present study provides clear evidence that baseline UA levels increase in malaria and that UA levels correlate with the levels of multiple cytokines implicated in the pathogenesis of this disease. Confirming a role for soluble UA in causing the symptoms and complications of malaria may require clinical trials of allopurinol or uricosuric drugs as adjunctive therapies. Immunohistochemical staining of autopsy specimens, or biopsies of muscle and dermis in live patients with uncomplicated *P. falciparum* malaria, may provide direct proof that parasite-derived UA precipitates are localized to microvessels and in contact with immune or other host cells (e.g., endothelial cells) that produce the cytokines we measured.
